# Editorial: Case reports in breast cancer : 2022

**DOI:** 10.3389/fonc.2023.1330225

**Published:** 2023-12-14

**Authors:** Nan Wang

**Affiliations:** Department of Breast Surgery, The First Affiliated Hospital of Zhengzhou University, Zhengzhou, China

**Keywords:** Pseudo-Meigs’ syndrome, tamoxifen-associated acute pancreatitis, pseudoprogression, PET/CT, Sacituzumab-govitecan, PARP inhibitors, IORT, ultrasound-guided microwave ablation

## 1 Introduction

Although case reports are considered to be at a lower level of evidence compared with systematic reviews, randomized controlled trials, and meta-analyses, in the history of medical development, the importance of clinical case reports cannot be ignored. Case reports also play an important role in the era of evidence-based medicine and have served as the building blocks of medical knowledge. These succinct narratives, grounded in real-world patient experiences, offer a rich tapestry of evidence that provided valuable insights into rare or atypical clinical scenarios, novel therapeutic approaches, and unexpected side effects. They have a special place in advancing the field of breast cancer (BC) care by shedding light on the nuances of diagnosis, treatment, and patient management. As guest editor for the Research Topic, “*Case Reports in Breast Cancer: 2022*,” I am delighted to present a summary of the nine insightful case reports that have contributed to our understanding of BC over the past year. In the pages that follow, we traverse the landscapes of clinical intricacies, therapeutic challenges, and diagnostic innovations presented by these case reports. Each narrative, a testament to the invaluable role of case reports in the fabric of evidence-based medicine, adds to the collective wisdom of our field.

## 2 Summary of case reports

Within this compendium, we embark on a journey through diverse clinical landscapes, offering glimpses into the complexity of BC. Two case reports delve into the clinical diagnosis and treatment of BC. The first report unravels the intriguing case of ovarian metastasis of BC, unveiling the clinical manifestation of pseudo-Meigs’ syndrome (Lin et al.). In contrast, the second report narrates the clinical intricacies of post-operative BC patients facing severe hyperlipidemia-induced acute pancreatitis following the administration of tamoxifen (Zhai et al.).

The cause of ascites in pseudo-Meigs’ syndrome remains unclear. It may be related to the stimulation of the peritoneum by hard solid ovarian tumors (1), or it may be related to the leakage caused by the pressure difference between the arterial supply of large tumors and the accompanying venous and lymphatic drainage (2), it may also be related to increased capillary permeability due to elevated intraperitoneal inflammatory cytokines and vascular endothelial growth factor (VEGF) (3). Cases of ovarian metastasis of BC with pseudo-Meigs’ syndrome is extremely rare. Among the reported cases of pseudo-Meigs’ syndrome caused by ovarian metastasis of BC, this report in our Research Topic is the fifth case (1). In the previous 4 cases and this case report, all patients with ovarian metastasis from BC complicated by pseudo-Meigs syndrome had estrogen receptor-positive (ER+) BC (4–7). I think this is very interesting and may be useful for studying BC metastasis to the ovary. Basic research on the relevant factors is suggestive, and I support and encourage clinicians to report such cases, which will make it easier for us to look for the same factors in different cases.

Tamoxifen is a medication commonly used in the treatment of hormone receptor-positive (HR+) BC, and it is known to have several potential side effects. One of the less common but significant side effects associated with long-term use of tamoxifen is an increase in triglyceride (TG) levels (8, 9), which, in rare cases, can lead to the development of acute pancreatitis (10–12). Acute pancreatitis is a serious and potentially life-threatening condition characterized by inflammation of the pancreas. The exact mechanism by which tamoxifen affects lipid metabolism is not fully understood, but it may involve alterations in the liver’s synthesis and secretion of TG, as well as changes in lipid clearance from the bloodstream. If a patient on tamoxifen develops hypertriglyceridemia or acute pancreatitis (13), treatment typically involves discontinuation of tamoxifen and management of the underlying condition (14). In this case report of tamoxifen-induced hypertriglyceridemia and acute pancreatitis (Zhai et al.), the authors used a comprehensive treatment plan including fasting, strict non-fat total parenteral nutrition, insulin combined with subcutaneous injection of low molecular weight heparin, etc. The plasma total TG of the two patients was quickly and effectively reduced to normal within 3 to 6 days after admission, thereby avoiding hemodialysis and plasma exchange. The method adopted by the authors is simple, cheap, safe, effective, easy to promote clinically, and is worth learning from.

Response Evaluation Criteria in Solid Tumors version 1.1 (RECIST Ver1.1) has been widely adopted in clinical trials and clinical practice to evaluate the treatment effect of solid tumors, including BC (15). RECIST V1.1 is mainly based on computed tomography (CT) to evaluate the patient’s condition. Although the European Organization for Research and Treatment of Cancer (EORTC) developed recommendations for ^18^F-fluorodeoxyglucose positron emission tomography (^18^F-FDG PET) (16) and PET response criteria for solid tumors (17) ^18^F-FDG PET has failed to become a standard for response assessment in solid tumors (18). F-fluoroestradiol (^18^F-FES) is a specific ER-targeting molecular probe used for PET assessment of ER expression in cancer (18) ^18^F-FES uptake is closely related to human estrogen receptor-α (ERα) immunohistochemistry (IHC) score, clinical studies have shown that ^18^F-FES PET can reliably detect ER+ BC lesions (19, 20). Research findings have demonstrated that ^18^F-FES PET is capable of assessing the extent of metastatic heterogeneity and furnishing valuable prognostic information regarding overall survival (OS) when evaluating bone lesions in patients with ER+BC at the time of their initial diagnosis (21). A retrospective head-to-head study comparing the efficacy of 18F-FES and 18F-FDG PET/CT in the context of metastatic invasive lobular BC revealed that 18F-FES PET demonstrated effectiveness in pinpointing metastatic sites, with a particular advantage observed in identifying bone metastases over 18F-FDG PET (22).

In this Research Topic, we have collected three interesting case reports related to PET/CT. Two of these reports underscore the remarkable sensitivity of ^18^F-FDG PET in assessing treatment response in patients with advanced BC, reaffirming the importance of this imaging modality in monitoring therapeutic efficacy. In a case report, a patient with advanced metastatic breast cancer (MBC) and multiple bone metastases had relief of bone pain symptoms after treatment, but the efficacy evaluation of conventional imaging (CT and SPECT) suggested that her disease had progressed. Fortunately, she finally confirmed the remission of her disease through ^18^F-FDG PET/CT examination (Tian et al.). In another case report, when evaluating the response of liver metastases to treatment in a patient with human growth factor receptor 2 (HER2) positive MBC, the efficacy assessment based on ^18^F-FDG PET examination reflected the treatment response more accurately than the efficacy assessment based on CT examination (Suto et al.). In the third case report ^18^F-FES PET non-invasive demonstrated the ER heterogeneity of tumors in a ER+/HER2-MBC patient and predicted the efficacy of using second and third line cyclin-dependent kinase 4/6 (CDK4/6) inhibitors after treatment failure with first-line CDK4/6 inhibitor (Pan et al.).

Triple-negative breast cancer (TNBC) is the most aggressive subtype of BC, and is known to be associated with a poor prognosis and limited therapeutic options. The breast cancer susceptibility genes (BRCA), specifically BRCA1 and BRCA2, play a crucial role in the occurrence and development of BC (23). These genes are involved in deoxyribonucleic acid (DNA) repair and help maintain genomic stability (24). Poly (ADP-ribose) polymerase (PARP) inhibitors are targeted therapeutics that have demonstrated efficacy as monotherapy in metastatic BRCA-mutant (BRCA-MUT) TNBC patients (25). We had incorporated two case reports that pertain to the systemic treatment of MBC in individuals with BRCA gene mutations in this Research Topic. These reports individually chronicle the treatment strategies employed—one involving the utilization of antibody-conjugated drugs and the other involving the administration of PARP inhibitors—and both achieved notable therapeutic outcomes (Mauro et al., Albarran et al.).

One of the case reports included in this Research Topic focuses on the treatment of a patient with active brain metastases (BMS) due to BRCA-mutant triple-negative MBC using a combination of Sacituzumab-govitecan (SG) and radiotherapy. In Italy, the use of PARP inhibitors for the treatment of MBC is allowed only after platinum-based chemotherapy failure. The MBC patient with a BRCA2 germline mutation experienced rapid brain metastases after only 3 months of first-line treatment. Since the major clinical trials of PARP inhibitors excluded platinum-refractory disease and their intracranial activity remained uncertain, the authors ultimately opted for SG combined with whole-brain radiotherapy as a second-line treatment for this patient. This patient’s progression free survival (PFS) (10 months) was significantly better than the median PFS (2.8months) for TNBC patients with brain metastases in the pivotal trial (26, 27). This case report provides support for the potential role of SG in treating active BMS in patients with BRCA-mutant TNBCand offers reference data regarding the safety of combining SG with whole-brain radiation therapy (WBRT). In another case report, a BC patient with a pathogenic germline BRCA2 mutation experienced early disease relapse while receiving adjuvant therapy with tamoxifen. She refused to use endocrine therapy alone or in combination with CDK4/6 inhibitors as a first-line treatment after recurrence due to disappointment with endocrine therapy and opted for a treatment regimen of talazoparib, at a dosage of 1 mg/24 hours. The patient achieved a complete disease remission (CR) after two cycles and her condition remained stable until the last follow-up time before the publication (November 2022). This case report represents the longest reported response to a PARP inhibitor to date and the first long-term response reported with talazoparib. This case report highlights the potential of using PARP inhibitors as a first-line treatment option for patients with HR-positive/HER2-negative BC who have disease recurrence and carry a pathogenic germline BRCA2 mutation. It suggests the potential of PARP inhibitors in this specific patient population.

Additionally, two other reports within this Research Topic center on local treatment strategies for breast tumors. One report underscores the significance of intraoperative radiotherapy (IORT) as a standard approach for BC patients with disabilities or compromised health (Omosule et al.). The other presents a case report on ultrasound-guided microwave ablation of a benign giant breast leiomyoma. While the latter does not directly address malignant tumors, the data it provides can inform local treatment strategies for BC and holds potential significance in this regard (Zhang et al.).

Intraoperative radiotherapy offers a potential solution for older patients with BC who may have debilitating health conditions or impairments (28). This technique delivers a targeted dose of radiation directly to the tumor bed during surgery, reducing the need for prolonged external beam radiotherapy sessions. The case report on IORT for BC in an elderly patient holds significant importance in the context of BC treatment. This case report emphasizes the successful application of intraoperative radiotherapy in an older patient, demonstrating the feasibility and efficacy of this treatment modality (Omosule et al.). By shedding light on the benefits and outcomes of intraoperative radiotherapy in this specific patient population, this case report contributes to the expanding knowledge and understanding of personalized treatment options for older adults with breast cancer. It underscores the significance of tailoring therapeutic approaches to meet the unique needs and characteristics of elderly patients, ultimately improving their quality of life and treatment outcomes. Therefore, the insights gained from this case report are instrumental in guiding clinicians and researchers towards more patient-centered and evidence-based care for elderly BC patients, fostering advancements in the field of geriatric oncology.

In addition to these 8 BC-specific case reports, we also present a case report addressing ultrasound-guided microwave ablation (US-MWA) for a benign giant breast leiomyoma (Zhang et al.). US-MWA is a minimally invasive technique used for the treatment of various types of tumors, including malignant phyllodes tumors and soft tissue sarcomas of the breast. It is a thermal ablation method that uses microwave energy to heat and destroy cancerous tissues. When combined with ultrasound guidance, US-MWA becomes a precise and effective approach for treating these tumors. The authors used US-MWA in the treatment of a breast mass located in close proximity to the pectoralis major muscle, measuring in excess of 7 cm in diameter—a dimension unprecedented in the existing literature on ablation (29–31). To enhance the efficacy of the ablation procedure, the authors innovatively employed refrigerated sterile saline to create a protective barrier, isolating the tumor from adjacent tissues. This approach effectively mitigated the thermal effects of the ablation, resulting in reduced patient discomfort and improved surgical field visibility for the operating surgeon. Moreover, the surgical methodology incorporated strategic interventions, including the implementation of short-term and long-term intervals, as well as multiple ablations. These measures were meticulously executed during the procedure to optimize the safeguarding of surrounding tissues and muscle structures against thermal damage, thus further elevating the quality of care provided. While not directly focused on malignant tumors, this report offers reference data that could have implications for local treatment strategies in BC.

## 3 Conclusion

In this Research Topic, the cases we collected emphasized the significant sensitivity of ^18^F-FDG PET in evaluating the therapeutic response of late-stage BC patients, the predictive value of ^18^F-FES PET in ER+/HER2- MBC for CDK4/6 inhibitor treatment response, the clinical manifestations of pseudo-Meigs syndrome caused by BC ovarian metastasis, the clinical complexity of severe hyperlipidemia and acute pancreatitis in BC patients receiving tamoxifen treatment, and the remarkable therapeutic effects of antibody-drug conjugates and PARP inhibitors in the systemic treatment of BC patients with BRCA mutations. In terms of local treatment strategies for breast tumors, the cases we collected highlighted the importance of IORT as a standard approach for BC patients with disabilities or comorbidities, and the surgical experience of using US-MWA for the treatment of large breast tumors. Each of these case reports is a valuable piece of the BC puzzle, contributing to our evolving understanding of this complex disease.

## Author contributions

NW: Writing – original draft, Writing – review & editing.
